# An *in-silico* Investigation Into the Role of Strain and Structure on Vascular Smooth Muscle Cell Growth

**DOI:** 10.3389/fbioe.2021.641794

**Published:** 2021-04-20

**Authors:** Orla M. McGee, David R. Nolan, Pattie S. Mathieu, Caitríona Lally

**Affiliations:** ^1^Trinity Centre for Biomedical Engineering, Trinity College Dublin, Trinity Biomedical Sciences Institute, Dublin, Ireland; ^2^Department of Mechanical, Manufacturing & Biomedical Engineering, School of Engineering, Trinity College Dublin, Dublin, Ireland; ^3^Advanced Materials and Bioengineering Research Centre (AMBER), Royal College of Surgeons in Ireland & Trinity College Dublin, Dublin, Ireland

**Keywords:** agent-based model, mechanobiology, collagen, vascular smooth muscle cells, stretch-avoidance, reorientation

## Abstract

The orientation of vascular cells can greatly influence the *in vivo* mechanical properties and functionality of soft vascular tissues. How cell orientation mediates the growth response of cells is of critical importance in understanding the response of soft tissues to mechanical stimuli or injury. To date, considerable evidence has shown that cells align with structural cues such as collagen fibers. However, in the presence of uniaxial cyclic strain on unstructured substrates, cells generally align themselves perpendicularly to the mechanical stimulus, such as strain, a phenomenon known as “strain avoidance.” The cellular response to this interplay between structural cues and a mechanical stimulus is poorly understood. A recent *in vitro* experimental study in our lab has investigated both the individual and collective response of rat aortic smooth muscle cells (RASMC) to structural (collagenous aligned constructs) and mechanical (cyclic strain) cues. In this study, a 2D agent-based model (ABM) is developed to simulate the collective response of RASMC to varying amplitudes of cyclic strain (0–10%, 2–8%, 4–6%) when seeded on unstructured (PDMS) and structured (decellularized collagenous tissue) constructs. An ABM is presented that is fit to the experimental outcomes in terms of cellular alignment and cell growth on PDMS substrates, under cyclic strain amplitudes of (4–6%, 2–8%, 0–10%) at 24 and 72 h timepoints. Furthermore, the ABM can predict RASMC alignment and change in cell number on a structured construct at a cyclic strain amplitude of 0–10% after 10 days. The ABM suggests that strain avoidance behavior observed in cells is dominated by selective cell proliferation and apoptosis at these early time points, as opposed to cell re-orientation, i.e., cells perpendicular to the strain increase their rate of proliferation, whilst the rate of apoptosis simultaneously increases in cells parallel to the strain direction. The development of *in-silico* modeling platforms, such as that presented here, allow for further understanding of the response of cells to changes in their mechanical environment. Such models offer an efficient and robust means to design and optimize the compliance and topological structure of implantable devices and could be used to aid the design of next-generation vascular grafts and stents.

## Introduction

Cardiovascular disease is the leading cause of death in the US and is attributed to one in every three deaths (Benjamin et al., 2017). Stenting is the most common treatment for stenosed arteries, with over one million procedures performed annually in the US alone (Benjamin et al., 2019). However in-stent restenosis occurs in 5–10% of cases (Byrne et al., 2015). Interventions such as stenting can change the arrangement of collagen fibers within the vessel. A study carried out on sheep found that after implanting self-expanding heart valve stents, regional differences were found in the collagen organization with collagen fibers near the strut aligning in the direction of the strut and random collagen orientation observed between struts (Ghazanfari et al., 2016). These changes in the collagen alignment alter the cell environment and the structural cues experienced by the cells. Previous research has demonstrated how mechanical and structural cues regulate the alignment of cells and collagen *in vitro* (Dickinson et al., 1994; Lee et al., 2008; Melvin et al., 2011; Rouillard and Holmes, 2012), and there is a need to better understand how cells integrate these cues as they remodel the extracellular matrix in biological processes like in-stent restenosis (Nolan and Lally, 2018), vascular graft repopulation (Zahedmanesh and Lally, 2012), and wound healing (Rouillard and Holmes, 2012). To date, research has shown that the orientation of cells greatly influences the *in vivo* mechanical properties and functionality of soft tissues (Ristori et al., 2018). In particular, cells excrete collagen along their primary direction (Sawhney and Howard, 2002; Wang et al., 2003; Matsugaki et al., 2013). As collagen is the main load-bearing component (Ristori et al., 2018), an understanding of cellular orientation is of huge importance in understanding the response of soft tissues to mechanical stimuli or injury. Previous experimental studies have demonstrated that cells align to topographical cues provided by collagen (Guido and Tranquillo, 1993) with numerous studies demonstrating that cells seeded in differentially constrained collagenous constructs align in the direction of the constraints (Huang et al., 1993; Thomopoulos et al., 2005; Henshaw et al., 2006; Foolen et al., 2012, 2018; Ristori et al., 2018). However, in the presence of uniaxial or cyclic strain, cells prefer to align themselves perpendicularly to the mechanical stimulus, known as “strain avoidance” (Foolen et al., 2012, 2014; de Jonge et al., 2013). Further to this, the ability of cells to reorient in response to mechanical stimuli is dependent on the density of the collagen fibers (Foolen et al., 2012, 2014). This interplay between collagen fibers and cells is not yet fully understood with further research required (Foolen et al., 2012; Ristori et al., 2018). A study by Rouillard and Holmes (Rouillard and Holmes, 2012) developed an agent-based model (ABM) to examine healing in infarcts that included structural, mechanical and chemical cues. This study, however, did not include strain avoidance demonstrated by cells under cyclic loading conditions.

**FIGURE 1:**
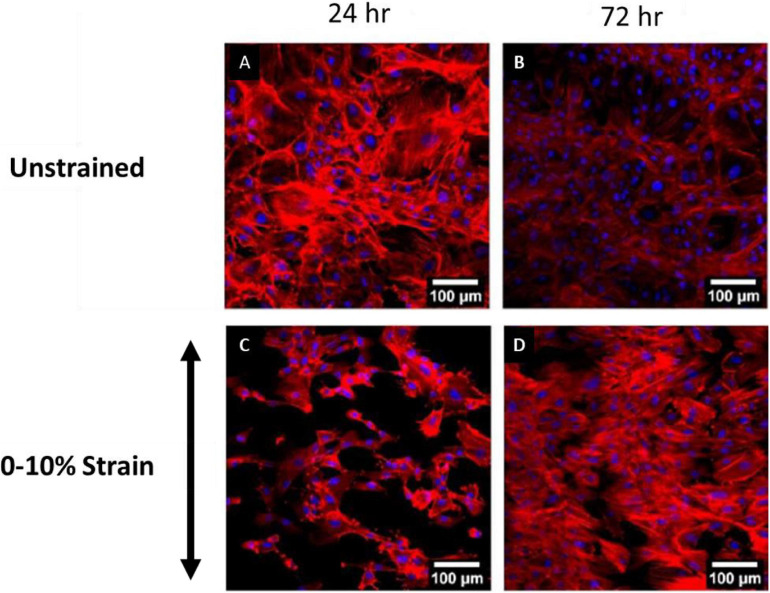
Images of RASMC cultured on PDMS after 24 h **(A,C)** and 72 h **(B,D)** of no strain **(A,B)** or 0–10% 1 Hz cyclic tensile strain **(C,D)**. Blue—DAPI nuclei, Red—Phalloidin f-actin (Mathieu, 2020).

**FIGURE 2:**
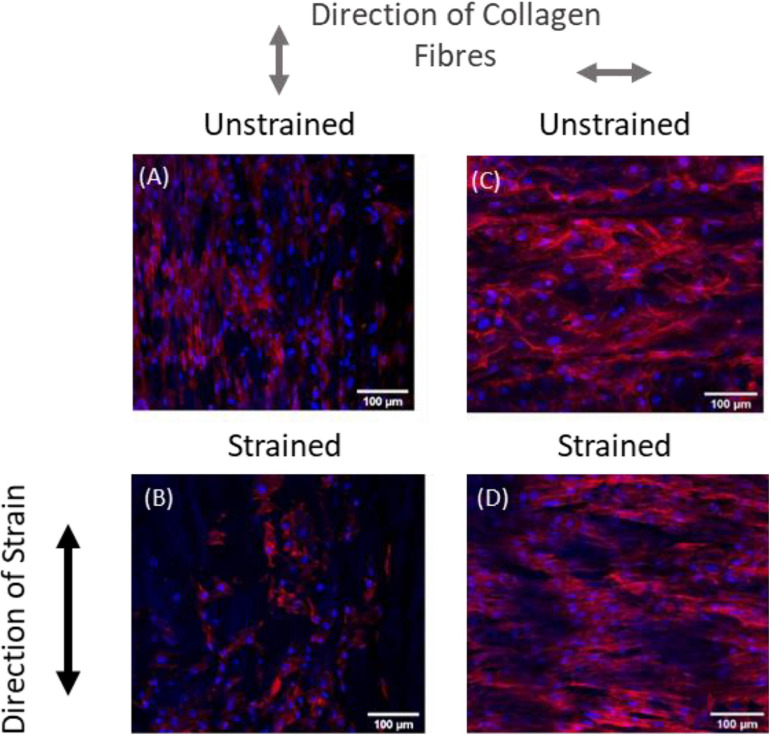
Representative images of RASMC on decellularized porcine carotid artery samples **(A–D)** left unstrained, **(A,C)** or strained parallel **(B)** or perpendicular **(D)** to the direction of collagen fibers. Blue—Nuclei, Red—F-actin (Mathieu, 2020).

Recent experimental studies in our lab examined the strain response of Rat Aortic Smooth Muscle Cells (RASMC) to varying amplitudes of cyclic strain on both unstructured dimethylpolysiloxane (PDMS) (Figure 1) substrates (Mathieu et al., 2020) and on decellularized collagenous tissue substrates (Figure 2) which presented topographical cues to the cells (Mathieu, 2020). It was found that RASMC seeded on PDMS exhibited a strain avoidance response that increased as the strain amplitude increased (additional experimental data can be found in the Supplementary Material). It was also found that RASMC strain avoid to a greater degree after 72 h. Furthermore, a strain-induced decrease in cell number was observed for the RASMC (Mathieu et al., 2020). When seeded on decellularized collagenous tissue substrates RASMC aligned with the topographical cues in two of the four samples while two samples exhibited strain avoidance behavior. The cells that remained aligned parallel to the strain direction showed a greatly reduced cell number in compared to the samples in which cells reoriented perpendicular to the strain direction. Figure 2 demonstrates that cells aligned with the direction of collagen fibers irrespective of the direction of strain (Mathieu, 2020).

The objective of this study is to investigate the combined response of cells to both structural and mechanical cues using a numerical framework and verify this framework using this aforementioned experimental data. Combining these agent-based modeling techniques with the existing experimental data allows us to generate a greater understanding of cellular behavior. This work aims to develop a modeling framework that can be used to investigate this response in a manner which cannot be assessed *in vitro/in vivo*.

## Materials and Methods

A 2D ABM was developed in MATLAB (R2018b, MathWorks) to simulate the collective response of RASMC to varying amplitudes of cyclic strain (0–10%, 2–8%, 4–6%) when seeded on an unstructured (PDMS) and a structured (decellularized collagenous tissue) substrate. It is known that smooth muscle cells are spindle-like in shape (Zhou et al., 2018). However, in this model, each individual vascular smooth muscle cell (VSMC) was represented as a circle agent with a radius, *R_c* and the cell direction is defined by a vector with angle, θ_*cell*,*t*_. *In vitro* and *in vivo* cells can move and change their shape to accommodate cells around them in highly dense populations. Cells in this model will only proliferate if there is space to do so. It was found that using a cell radius of 12.96 μm (Zahedmanesh and Lally, 2012; Zahedmanesh et al., 2014) lead to artificial over confluence at high cell densities. Therefore, to accurately represent the cell turnover at higher cell densities a value of 0.3888 μm was chosen as *R_c*. Further information on this can be found in the Supplementary Material.

VSMC agents were seeded randomly as a monolayer on 2D constructs representing PDMS and decellularized collagenous tissue based on (Mathieu et al., 2020) and (Mathieu, 2020) with seeding densities of 5.5 × 10^3^ cells/cm^2^ and 1.33 × 10^4^ cells/cm^2^ on each of the respective constructs. Each VSMC agent was then randomly assigned an angle between $\frac{\pi }{2}$ and $-\frac{\pi }{2}$ radians.

### Cell Reorientation Algorithm

An external cyclic strain was applied divided into its x, y and shear components. The mean strain, $\bar{\varepsilon }$, and the strain amplitude, △ε, were calculated using a Mohr’s circle approach. The maximum (ε_*max*_) and minimum (ε_*min*_) principal strains and the direction of the maximum principal strain ($\bar{\theta_{\text{p}}}$) were calculated from both △ε and $\bar{\varepsilon }$ respectively, using Mohr’s circle. The maximum principal strain amplitude (ε_*max*_) was assumed to be the strain amplitude experienced by the cell while the direction of principal strain is represented by $\bar{\theta_{\text{p}}}$. Based on previous studies (Zhu et al., 2011; Mathieu et al., 2020) demonstrating strain avoidance the cell strain avoid direction (θ_*csa*_) was calculated as:

$$\theta_{c\,s\,a}=\bar{\theta_{\text{p}}}+\frac{\pi }{2}$$

**FIGURE 3:**
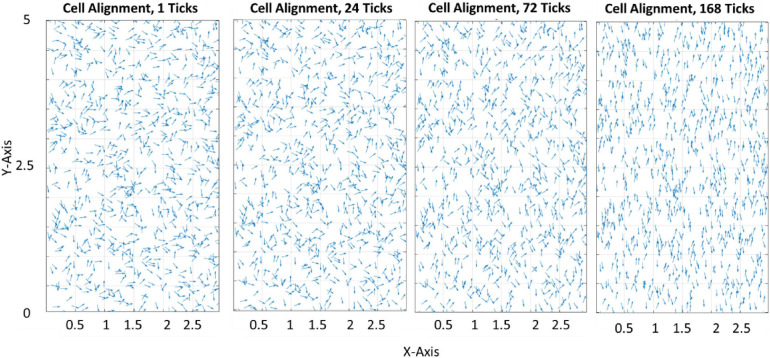
Quiver plot demonstrating the change in orientation of cells in response to 0–10% strain predicted by the model (pre-calibration to experimental data) without the inclusion of apoptosis or proliferation.

Figure 3 demonstrates how the model predicts cell reorientation over time in the absence of apoptosis or proliferation. It must be noted that as apoptosis and proliferation are not present this is not representative of a physiological phenomenon and cannot be calibrated to a group of cells. Therefore, ticks are used as a phenological representation of time in the model in lieu of a unit of time.

### Structural Cues (Fibers)

**FIGURE 4:**
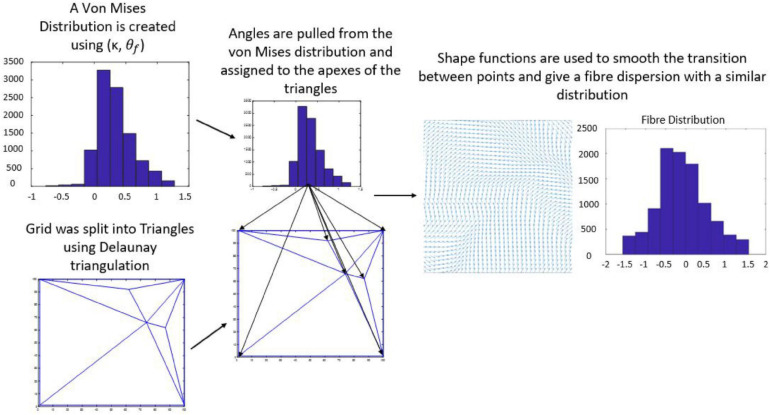
Flow chart demonstrating workflow used to create a fiber distribution.

**FIGURE 5:**
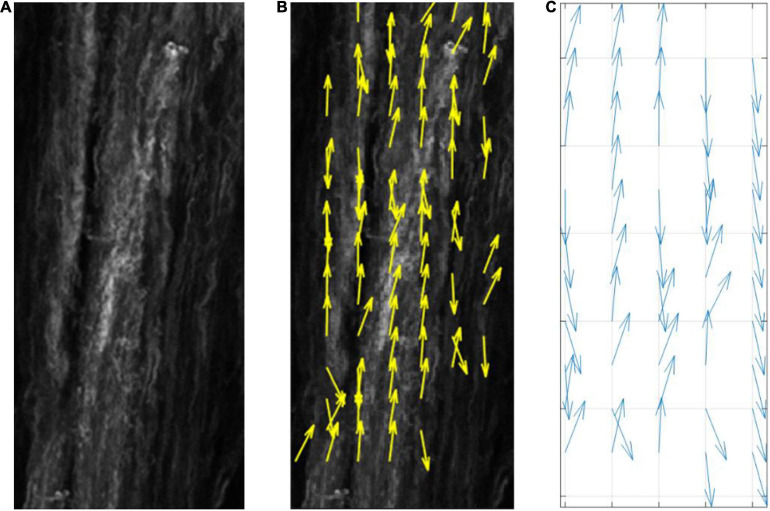
**(A)** Collagenous arterial tissue, **(B)** an image tracking the fiber orientation in the tissue created using MatFiber (Fomovsky and Holmes, 2010), **(C)** a representative distribution created by the model.

The average direction of the bundle of fibers (${\bar{\theta }}_{f\,b})$ was input into the model as parallel or perpendicular to the strain direction. A fiber dispersion term (κ) was also assigned to describe the dispersity of fibers on the 2D patch of tissue. The 2D patch was assigned a fiber pattern based on the average fiber direction. A random von Mises distribution was created with a mean direction, ${\bar{\theta }}_{f\,b}$ and the concentration parameter κ, using the circular statistics toolbox function *circ_vmrnd* (Berens, 2009). Using Delaunay triangulation, the grid was split into triangles with a number, *n* of vertices. A corresponding number, *n* of values were pulled from the von Mises distribution and each of the values were assigned to each vertex of the triangles. Shape functions were then used to interpolate fiber angle at given points within the triangles to give a smooth continuous distribution of fibers with a known mean angle and distribution (Figure 4), similar to distributions seen in biological tissue (Figure 5).

### Combined Mechanical and Structural Response

**FIGURE 6:**
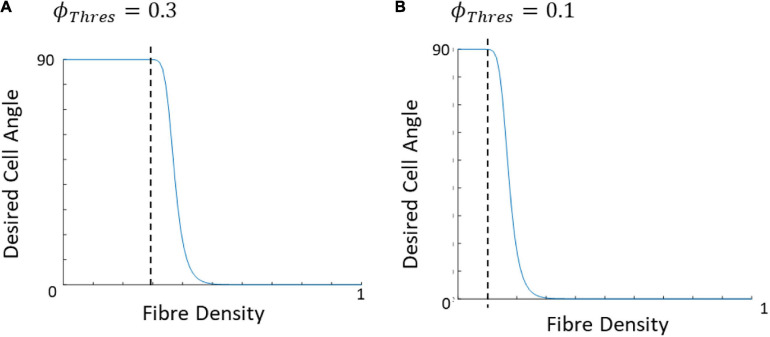
Graphical representation of the influence of ϕ_*Thres*_ on the desired cell angle, assuming both the strain and fiber direction are at 0°, when **(A)** ϕ_*Thres*_ is 0.3 and **(B)** ϕ_*Thres*_ is 0.1.

**FIGURE 7:**
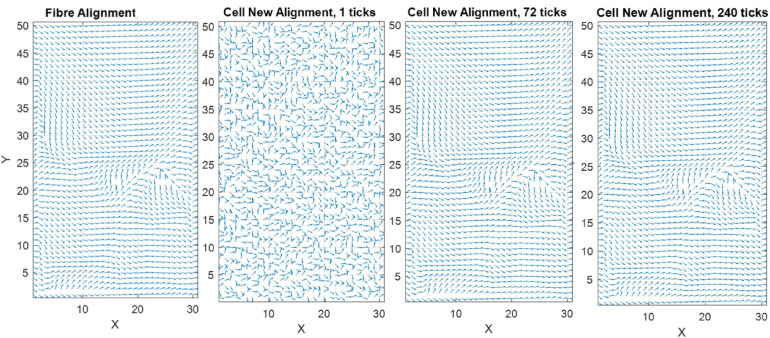
Quiver plot demonstrating the change in orientation of cells, in response to the influence of fibers alone, predicted by the model (pre-calibration to experimental data) without the inclusion of apoptosis or proliferation.

Once the fiber directions and the cell strain avoidance angles were calculated the combined response of cells to both mechanical and structural cues was calculated using:

$$\theta_{c\,e\,l\,l,f\,i\,n\,a\,l}=\theta_{f}+\frac{\left(\theta_{c\,s\,a}-\theta_{f}\right)}{1+M^{10\,\left(\phi_{f}-\phi_{T\,h\,r\,e\,s}\right)}}$$

where θ_*cell*,*final*_ is the angle the cell desires to achieve or final cell angle, θ_*f*_ is the angle of the closest corresponding fiber to the cell, ϕ_*f*_, and is a linear (0–1) representation of the density of the fibers, 1 being where the fibers dominate the cell direction and 0 meaning strain is dominant and there are no fibers present. The ϕ_*Thres*_ is the point at which fibers begin to influence cell reorientation (Figure 6) while *M* controls the slope of the curve. Figure 7 demonstrates cellular response to fibers alone in the absence of strain.

It was assumed the speed at which the cells reorient in response to structural and mechanical cues differ. This was represented in the model using the ratio of ϕ_*f*_ to the ϕ_*Thres*_ as an indicator for the rate at which a cell reorients whereby:

For ϕ_*f*_ < ϕ_*Thres*_:

$$\delta \,\theta_{c\,e\,l\,l}=Z_{e\,f\,f}\,K_{r\,o\,t,c}$$

For ϕ_*f*_ > ϕ_*Thres*_:

$$\delta \,\theta_{c\,e\,l\,l}=\left|s\,i\,n\,\left(\theta_{f}-\theta_{c\,e\,l\,l,t}\right)\right|\,K_{r\,o\,t,f}$$

where θ_*cell*,*t*_ is the current cell angle in radians and *K*_*rot,c*_ and *K*_*rot,f*_ are variables that are calibrated to each individual cell type that dictate the rate at which cells reorient for a given time point. *Z*_*eff*_ is the effective stimulus and refers to the amount of stimulus experienced by a given cell. This can be calculated using:

$$Z_{e\,f\,f}=\frac{\varepsilon_{e\,f\,f}-\varepsilon_{T\,h\,r\,e\,s}}{\varepsilon_{M\,a\,x}-\varepsilon_{T\,h\,r\,e\,s}}$$

whereby, ε_*Max*_ is the maximum strain that will influence reorientation. Above this, a cell is already reorienting as quick as possible. ε_*Thres*_ is the threshold of strain below which the strain is too low to influence the cell reorientation. ε_*eff*_ is the effective strain i.e., the strain experienced by the cell based on its orientation and can be described by:

$$\varepsilon_{e\,f\,f}=\Delta \,\varepsilon \,x^{\prime }\,\cos\alpha_{c\,p}$$

$$\alpha_{c\,p}=\theta_{c\,e\,l\,l,t}-\bar{\theta_{\text{p}}}$$

A direction (*Dir*_*c*_) of reorientation was assigned to each individual cell to ensure the cell took the shortest path from its current orientation to its desired orientation. *Dir*_*c*_ was assigned based on the value of the angle β_*dc*_, the difference in the current cell angle (θ_*cell*,*t*_) and the desired cell angle (θ_*cell*,*final*_) whereby:

$$\beta_{d\,c}=\theta_{c\,e\,l\,l,f\,i\,n\,a\,l}-\theta_{c\,e\,l\,l,t}$$

and if:

$$\beta_{d\,c}\geq 0\,D\,i\,r_{c}=1$$

$$\beta_{d\,c}<0\,D\,i\,r_{c}=-1$$

The new cell angle was calculated using:

$$\theta_{c\,e\,l\,l,t+1}=\theta_{c\,e\,l\,l,t}+D\,i\,r_{c}\,\delta \,\theta_{c\,e\,l\,l}$$

Upon developing an algorithm to predict cell reorientation based on mechanical and structural cues. The model was expanded to include apoptosis and proliferation of cells based on the mechanical stimulus experienced by individual cells.

### Apoptosis

As the cells were cultured *in vitro* it was assumed that they had a synthetic phenotype. The apoptosis rate was dictated by the value of cyclic strain experienced by the cells (Colombo, 2009). The probability of apoptosis was defined as a function of cyclic strain (Colombo, 2009; Zahedmanesh and Lally, 2012):

$$P_{A\,P}=A_{a\,p\,o\,p}\,\varepsilon_{c\,y\,c}+B_{a\,p\,o\,p}$$

where *P*_*AP*_ is the percentage probability of apoptosis and *A*_*apop*_, and *B*_*apop*_ were calibrated based on the experimental results. Based on the methods outlined in Zahedmanesh and Lally (2012) a logical statement was defined within each cell using a random number generator. If the statement was found to be true, the cell was removed from the simulation.

### Proliferation

Proliferation was modeled based on the doubling time for each individual cell in line with approaches previously used in Peirce et al. (2004) and Zahedmanesh and Lally (2012). The doubling time of each cell was defined for each individual cell based on a function of cyclic strain based on an experimental study by Colombo (Colombo, 2009) and the modeling framework present in Zahedmanesh and Lally (2012), described by:

$$T_{d}=A_{p\,r\,o\,l\,i\,f}\,\varepsilon_{c\,y\,c}^{2}+B_{p\,r\,o\,l\,i\,f}\,\varepsilon_{c\,y\,c}+C_{p\,r\,o\,l\,i\,f}$$

where *T_d* is the doubling time of a given cell and *A*_*prolif*_, *B*_*prolif*_ and *C*_*prolif*_ are constants calibrated to fit cellular alignment and cell growth on PDMS substrates, under cyclic strain amplitudes of (4–6%, 2–8%, 0–10%) at 24 and 72 h timepoints.

**FIGURE 8:**
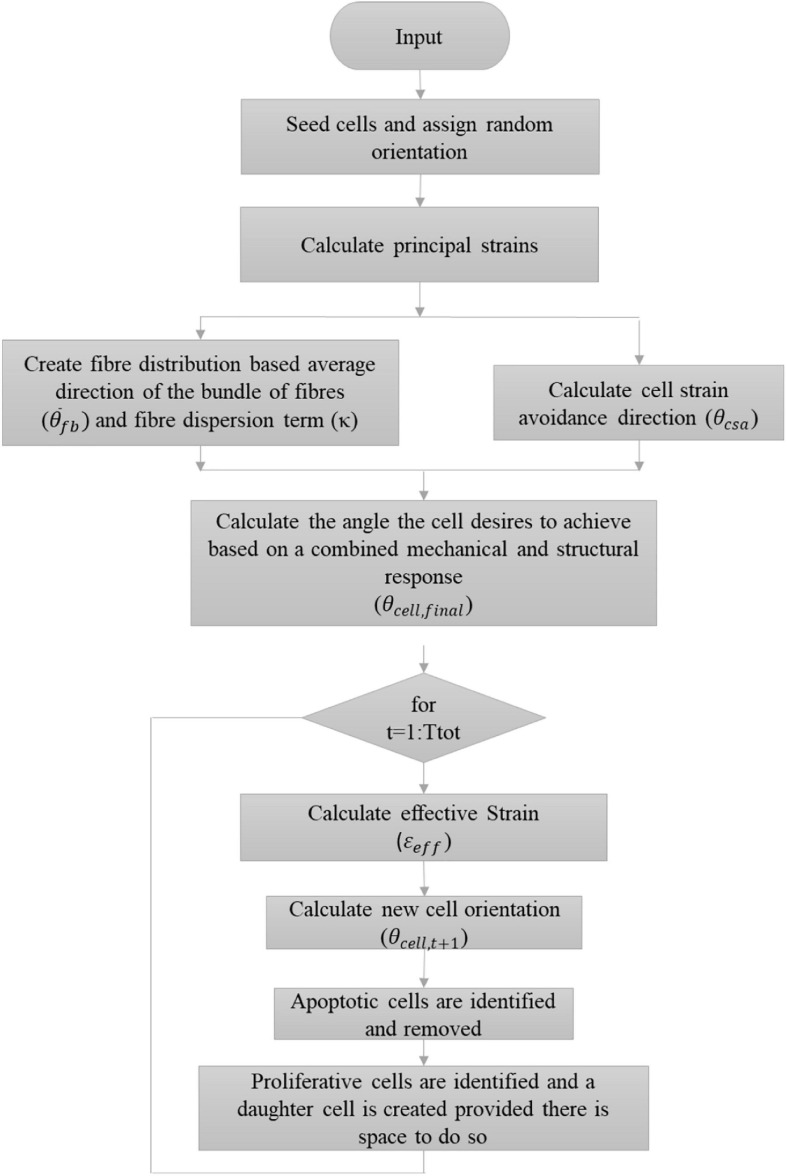
Flowchart describing the implemented algorithm.

Using methods developed in Nolan and Lally (2018) a cumulative Gaussian distribution function is then introduced to determine the probability of a cell to proliferate based on the cell age and the doubling time of a given cell calculated by:

$$P_{D\,T}\,\left(t_{a\,g\,e}\right)=\left(1+e\,r\,f\,\left[\frac{t_{a\,g\,e}-T_{d}}{\sqrt{2\,\sigma_{p\,r\,o\,l\,i\,f}^{2}}}\right]\right)$$

where *t*_*age*_ is the age of the VSMC agent, *T_d* is the doubling time of a cell with a standard deviation of σ_*prolif*_. This is a sigmoid shaped probability function where at age zero there is zero probability of cell proliferation and as the age of the cell increases so does its probability of proliferating until it is ultimately equal to 1. At the beginning of the model, each cell was randomly assigned an age between 0 and *C*_*prolif*_σ_*prolif*_. If it is determined that a cell should proliferate, a daughter cell of the same size may be created tangentially to the parent cell. The parent cell searches a full 360° range at 1° increment and checks whether a daughter cell may be created at that location without overlapping an existing cell. Once a list of angles where the daughter cell can successfully be produced without overlap is determined, a random number generator is used to pick a single location from the list where the daughter cell is ultimately created. The age of both the parent and daughter cells are set to 0. A more in-depth description of how the ABM determines the presence of surrounding cells can be found in Nolan and Lally (2018). Figure 8 demonstrates a flowchart further outlining the algorithm used.

### Parameter Calibration and Investigation

Once the model was set up it was used to understand the influence of the various parameters on the cellular response and compare the results of the model to the experimental data. The unknown parameters are listed in Table 5. The parameters were calibrated to experimental data examining the response to a) a control where no strain or structure was present and b) cyclic strain amplitudes of (4–6%, 2–8%, 0–10%). These parameters were then used to investigate the combination of cyclic strain and structure and compare the results to the experimental data of cells on parallel and perpendicularly aligned structured construct at a cyclic strain amplitude of 0–10% after 10 days.

#### Calibration to Response in the Absence of External Stimulus

Firstly, the influence of *B*_*apop*_, *C*_*prolif*_, and σ_*prolif*_ on cell behavior was investigated. These parameters investigated the response of cells in the absence of external stimuli. The parameters were compared and calibrated to the change in cell density of unstrained cells cultured on PDMS for 3 days (Table 1). As the strain was zero during this time the remaining unknown parameters were not involved in calculating the cell density at day 3.

**TABLE 1:** Change in cell density at day 3 (unstrained) (Mathieu, 2020; Mathieu et al., 2020).

Experimental cell density day 0 (cell/mm^2^)	Experimental day 3 (unstrained)	Model prediction day 3 (unstrained)
305.21 ± 204.64	682.81 ± 166.21	516.86
798.74 ± 379.51	1375.57 ± 810.3	1339.00
457.92 ± 70.875	746.11 ± 20.95	763.38

Based on (Endlich et al., 2000) where the doubling time of rat aortic VSMCs was found to be 71 ± 9 h, *C*_*prolif*_ and σ_*prolif*_ were set as 71 and 9, respectively. Once *C*_*prolif*_ and σ_*prolif*_ were assigned in this way, *B*_*apop*_ was calibrated using an iterative approach changing the value in increments of 0.01 and it was found that only a value of 1.64 could predict all three cell densities.

#### Calibration in Response to Cyclic Strain

Once the values *B*_*apop*_, *C*_*prolif*_ and σ_*prolif*_ were calibrated, the response of cells to cyclic strain when seeded on PDMS was modeled. The parameters relating to the influence of cyclic strain are outlined in Table 2.

**TABLE 2:** Parameters relating to cyclic strain.

Variable	Symbol
Strain threshold	ε_*Thres*_
Max strain	ε_*Max*_
Cell rotation constant	*k*_*rot,c*_
Apoptosis constant	*A*_*apop*_
Proliferation constants	*A*_*prolif*_
	*B*_*prolif*_

**FIGURE 9:**
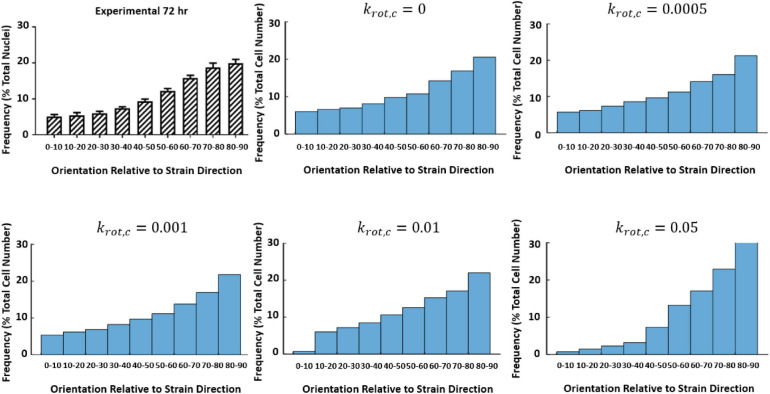
Influence of *k*_*rot*,*c*_ on cell reorientation (Mathieu, 2020; Mathieu et al., 2020).

An initial guess of *A*_*prolif*_, *B*_*prolif*_, *A*_*apop*_, ε_*Max*_, and ε_*Thres*_ that was found to accurately predict the fold change observed experimentally at 0–10% strain at 24 and 72 h was used to investigate the influence of *k*_*rot,c*_ (Figure 9).

Further investigation examining how the change of *k*_*rot*,*c*_influenced the fold change of the cells at 24 and 72 h time points can be found in Table 3. It was found a *k*_*rot,c*_ of 0.001 was most suitable in predicting cell behavior.

**TABLE 3:** The influence of *k*_*rot*,*c*_ on cell fold change.

*k*_*rot*,*c*_	Foldchange (72 h)
0	0.88
0.0005	0.92
0.001	0.88
0.01	0.91
0.05	1.03
Experiment	0.939 ± 0.12

The parameters ε_*Max*_ and ε_*Thres*_ were investigated examining the strain alone and it was found that a value of ε_*Max*_ of 0.4 and ε_*Thres*_ of 0.0 to the experimental data, details on these parameters are further expanded upon in the discussion. Once the influence of *k*_*rot,c*_ was understood and ε_*Max*_ and ε_*Thres*_ were defined the role of apoptosis and proliferation in response to cyclic strain was examined. The model was used to investigate all combinations of *A*_*prolif*_ from 0 to 3,500 in increments of 500, *B*_*prolif*_ from -100 to 500 in increments of 50 and *A*_*apop*_ from 0 to 20 in increments of 1 to predict fold change in cell number and compare the predicted values to the experimentally obtained fold change for the strained cells cultured on PDMS. Values of *A*_*prolif*_3,500, *B*_*prolif*_450 and *A*_*apop*_1 gave the best fit to the experimental data of cellular alignment and cell growth on PDMS substrates, under cyclic strain amplitudes of (4–6%, 2–8%, 0–10%) at 24 and 72 h timepoints. Table 4 shows the model predictions for fold change in cell number vs. the values observed experimentally.

**TABLE 4:** Fold change for cells at 24 and 72 h when seeded on PDMS and subject to different levels of cyclic strain (Mathieu et al., 2020).

	24 H		72 H	
Strain	Experimental	Model	Experimental	Model
4–6%	1.22 ± 0.19	1.44	1.34 ± 0.95	1.37
2–8%	0.99 ± 0.29	1.04	0.44 ± 0.15	1.08
0–10%	0.80 ± 0.14	0.87	0.939 ± 0.12	0.83

#### Cellular Response to Structure

**FIGURE 10:**
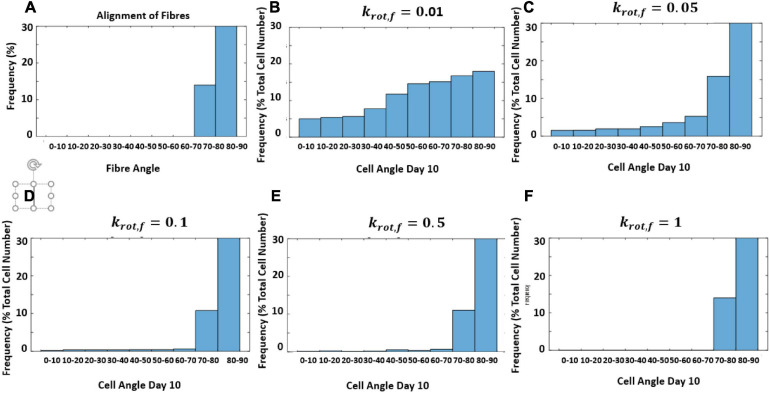
Histogram of predicted cell orientation after 10 days for different values of *k*_*rot*,*f*_ when seeded on the same distribution of fibers; **(A)** fiber angles in fiber distribution, **(B)** *k*_*rot*,*f*_ = 0.01 **(C)** *k*_*rot*,*f*_ = 0.05 **(D)** *k*_*rot*,*f*_ = 0.1 **(E)** *k*_*rot*,*f*_ = 0.5 **(F)** *k*_*rot*,*f*_ = 1.

In this model, it was not assumed that cells responded at the same rate to structure and strain. Therefore, the response rate of cells to structure without the influence of strain was investigated. The model is designed to create a new structure each time it runs based on an input of an average fiber direction using random von Mises distribution. This is done to account for variability in different pieces of structured decellularized tissue. However, to examine the influence of *k*_*rot,f*_ a constant fiber distribution was used in Figure 10A. Figure 10 shows the influence of changing *k*_*rot,f*_ on the reorientation of cells after 10 days as they align with the structure.

## Results

Model calibration found the following parameters (Table 5) to best fit the experimental data.

**TABLE 5:** Parameters calibrated against experimental data.

Variable	Symbol	Calibrated value
Threshold fiber density	ϕ_*Thres*_	Undefined
Slope	*M*	Undefined
Strain threshold	ε_*Thres*_	0.0
Max strain	ε_*Max*_	0.4
Cell rotation constant	*k*_*rot,c*_	0.001
Fiber rotation constant	*k*_*rot,f*_	0.1
Apoptosis constant	*A*_*apop*_	1
	*B*_*apop*_	1.64
Proliferation constants	*A*_*prolif*_	3,500
	*B*_*prolif*_	500
	*C*_*prolif*_	71 (Endlich et al., 2000)
	σ_*prolif*_	9 (Endlich et al., 2000)

### Predicting the Combined Response to Strain and Structure

Once the model was calibrated to the experimental data on PDMS substrates, under cyclic strain amplitudes of (4–6%, 2–8%, 0–10%) at 24 and 72 h timepoints it was used to predict the response of VSMC to both strain and structure and compare the results to those predicted in the experimental data investigating the fold change in cell number after 10 days of 0–10% cyclic strain when seeded on decellularized collagenous constructs aligned perpendicular and parallel to strain. Due to the fact that the levels of dispersion in the experimental collagenous constructs were unknown, the model was used to investigate different degrees of dispersion (κ); 2, 5, 8, 100. The fiber alignments generated for different variations of κ can be seen in the Supplementary Material. Table 6 shows the results of the model’s prediction for fold change after 10 days when aligned parallel and perpendicular to strain. It can be observed from the experimental data that cell fold change increases when cells are aligned perpendicular to the strain and decreases when cells are aligned with the direction of strain. This behavior is captured in the model. Furthermore, the model predicted that as fiber alignment increases fold change increases when fibers are perpendicular to strain and decreases when fibers are aligned parallel to strain.

**TABLE 6:** The fold change of cells a 0–10% cyclic strain when seeded on structured constructs aligned perpendicular or parallel to strain.

Fold change (10 days)	κ	Perpendicular	Parallel
Experiment	Unknown	1.23 ± 1.714	0.458 ± 0.035
Model	100	1.72	0.19
	8	1.28	0.21
	5	1.11	0.22
	2	0.72	0.48

## Discussion

In this study, a 2D ABM is presented to investigate the influence of cell behavior in response to cyclic strain and structure. This model was calibrated to experimental data of cell behavior to cyclic strain and used to predict cell growth when subjected to the combined influence of strain and structure. While the model was able to predict the trends in fold change in response to the combined response of strain and structure, it was somewhat limited by the lack of experimental data necessary to calibrate certain parameters in the model. By combining further experimental work with this model, a more robust design tool could be developed. This could be implemented to better understand cellular behavior and also as an efficient means to design and optimize the compliance and topological structure of implantable devices. Furthermore, a model such as this with further experimental calibration could be used to aid the design of next-generation vascular grafts and stents. Below is a comprehensive discussion of each of the model parameters, how they individually influence cellular response and suggestions for further experimentation that could lead to a more accurate representation of cellular behavior.

The first assumption of the model was a reduction in cell radius that is found for RASMC in literature. Using the RASMC cell radius value from literature it was found that the model could not predict the high cell densities seen in the experimental data. This is due to the fact that *in vitro* cells can change their shape to conform to cells around them. However, in the model cells were a strict circular shape and could not conform. It was observed at higher cells densities even though the total free area on the construct would allow for further proliferation, due to the rigidity of the cell shape in the model there was not adequate space for cells to double. Therefore, the model would achieve confluence earlier than the experimental data. Performing a sensitivity analysis, it was found that a cell radius of 0.3888 μm allowed proliferation to higher cell densities. Further information on this can be found in the Supplementary Material.

Another assumption was *C*_*prolif*_ was chosen based on the experimental study (Endlich et al., 2000). However, doubling time is calculated as the time it takes cells to double in number and consequently the balance between *B*_*apop*_ and *C*_*prolif*_ needs to be carefully considered. This ratio of cell death to cell growth will ultimately dictate the final cell density. Although understanding the ratio of cell death to growth can give an insight to cell behavior, further studies are required to understand the influence of these two parameters individually. Using stains like Ki67 as in Mathieu et al. (2020) could give further insight into the degree of cell proliferation occurring, however, Ki67 stains for a section of the mitosis cycle, whereas in the ABM mitosis is modeled as instant cell division making markers like this difficult to use for calibration. Future studies into the degree of proliferation and cell death occurring at hourly time points may give further insight into the individual influence of these two parameters on the collective behavior of this cell population. Furthermore, the mechanics of PDMS and collagenous tissue are different and this must be noted as substrate stiffness can influence cell properties leading to increased proliferation, migration and decreased cell contractility (Dieffenbach et al., 2017; Yi et al., 2019). However, the levels of strain induced are comparable between the two materials and as most variables were calibrated to the change in strain, the only assumption made between the two different substrates is that the cells had a similar doubling time and rate of apoptosis when left unstrained. Given the cells are the same cell type and this parameter was based on values taken from literature the authors feel this is a reasonable assumption.

Once the ABM performance had been calibrated by comparison to experimental data of cells without any strain or structural stimulus the model was used to investigate how the cells responded to cyclic strain. The rate of response of cells to cyclic strain (*k*_*rot,c*_) is a factor that the model has shown to be key in representing the correct reorientation of the cells. The model suggests that although cell reorientation appears to influence the overall cell alignment there is also a bias of cells proliferating in areas of low cyclic strain and cell death occurring in areas of high cyclic strain. Therefore, more apoptosis and less proliferation inherently occur in areas where cells are aligned with strain and conversely, less apoptosis and more proliferation occur where cells are aligned perpendicular to strain. One of the most interesting findings of this model is that even in the absence of cell reorientation (i.e., *k*_*rot*,*c*_0) we can still see that when predicting the same fold change as seen in the experimental data we observe similar reorientation plots due to the effective strain-based bias of apoptosis and proliferation. This finding leads the authors to two hypotheses (1) that cell reorientation is not occurring to any significant level in these experiments and that the “reorientation” observed is due to a greater extent to the bias of effective strain, or (2) that the rate of cell reorientation does not dominate the final cell position and it is the bias of effective strain and cells slowly reorienting combined that give us this overall result of cells aligned perpendicular to strain. Further experimental studies such as live-cell images of cell reorientation in response to cyclic strain could answer these questions concerning the rate of cell reorientation more definitively. Understanding the rate of cellular response and how cells respond to cyclic strain can have a huge impact on phenomena such as in-stent restenosis and other cascading events. Further insight into whether cell reorientation itself or whether the effective strain bias is dominating the beginning of this cellular response could provide further insight into these phenomena.

Additionally,*k*_*rot,f*_ could be investigated using a similar live-cell imaging approach to assess the rate of response of cells to structure. Currently, in this model depending on the strain rate the cells reorient at 0.007° per hour in response to strain and 2.8° per hour in response to structure, however, this would need to be verified through further experimentation examining more regular time points. Furthermore, the influence of fiber dispersion can be observed in the model, it was predicted that with an increase in fiber alignment, fold change increases when fibers are perpendicular to strain and decreases when fibers are aligned parallel to strain. This can be explained due to the fact that an increase in overall fiber alignment would lead to a greater number of cells aligning in the predominant fiber direction meaning in the case of perpendicular collagen alignment a greater proportion of cells would experience less strain leading to decreased apoptosis and increased proliferation generating an increase in foldchange. The opposite would be seen when collagen is aligned with strain, that is, a greater proportion of cells would experience more strain and therefore increased apoptosis and decreased proliferation would occur leading to the lower fold change as predicted in the model. The alignment and dispersion of the construct prior to cell seeding and cell alignment should be examined in future experiments. In the results shown here, a dispersion of κ between 2 and 5 seems most likely for the experimental constructs, however, this would need to be quantified.

In this model some terms were included that could not be calibrated from the existing experimental data and would require further experimental research to be able to accurately quantified. Firstly, the model contains a threshold fiber density parameter, ϕ_*Thres*_, and *M*, the slope, that dictates the density at which fibers begin to influence cellular behavior over other cues like cyclic strain. This may vary from cell type to cell type depending on the size of the cells relative to the space between fibers on a structured construct. In the model, ϕ_*Thres*_ and *M* are used to represent the point at which fibers begin to dominate the influence of cell behavior based on the normalized cell density (0–1) where 0 means no fibers are present and 1 represents a dense collagenous tissue. In the model, it was assumed PDMS has a fiber density of zero and collagen tissue has a fiber density of 1. Therefore, in the experiments used to calibrate the model ϕ_*Thres*_ and *M* cannot be quantified and remain undefined. These parameters were included to allow for the improvement of the model with further experimentation. Experiments with different structured constructs of different density are necessary to calibrate these parameters. Furthermore, the maximum strain that can influence cell behavior, ε_*Max*_, was briefly examined in the paper and assigned a value of 0.4, however, experiments examining higher levels of strain must be carried out to quantify this parameter. Similarly, to correctly identify the threshold strain, ε_*Thres*_, lower levels of cyclic strain must be examined experimentally.

Finally, the influence of *A*_*prolif*_,*A*_*apop*_, and *B*_*prolif*_ are parameters that are challenging to quantify experimentally as separating the influence of proliferation and apoptosis in cellular experiments is very difficult. The calibration shown does not capture the experimental data at all three levels of cyclic strain. However, having a better understanding of other parameters in the model would lead to increased accuracy in the prediction of these parameters and a better understanding of cell behavior in relation to cyclic strain.

## Conclusion

In conclusion, this paper presents an ABM that can be used to predict cell behavior in response to cyclic strain and structural cues. The model was calibrated against existing experimental data and used to predict the fold change in cell number after 10 days of 0–10% cyclic strain when seeded on structured constructs aligned perpendicular and parallel to the direction of cyclic strain. The model successfully captured the trends in cell growth seen experimentally. This model gives insight into the role of different factors influencing cells that cannot easily be examined experimentally, such as highlighting the role of effective strain bias in influencing cell alignment. Tools such as this ABM can be used in conjunction with in vitro experimental data to further enhance the understanding of cellular behavior in response to intravascular medical devices.

## Data Availability Statement

The raw data supporting the conclusions of this article will be made available by the authors, without undue reservation.

## Author Contributions

OM was the primary researcher and developed the model. DN contributed to the model and its development. PM provided the experimental data referenced here and used in the manuscript. CL was the principal investigator who conceived the study and provided insight into the model. All authors contributed to writing and editing the manuscript.

## Conflict of Interest

The authors declare that the research was conducted in the absence of any commercial or financial relationships that could be construed as a potential conflict of interest.

## References

[B1] Benjamin E. J. Blaha M. J. Chiuve S. E. Cushman M. Das S. R. Deo R. (2017). Heart disease and stroke statistics-2017 update: a report from the American Heart Association. *Circulation* 135, e146–e603. 10.1161/CIR.0000000000000485 28122885 PMC5408160

[B2] Benjamin E. J. Muntner P. Alonso A. Bittencourt M. S. Callaway C. W. Carson A. P. (2019). Heart disease and stroke statistics-2019 update: a report from the American Heart Association. *Circulation* 139 e56–e528. 10.1161/CIR.0000000000000659 30700139

[B3] Berens P. (2009). CircStat: a MATLAB toolbox for circular statistics. *J. Stat. Softw.* 31 1–21. 10.18637/jss.v031.i10

[B4] Byrne R. A. Joner M. Kastrati A. (2015). Stent thrombosis and restenosis: what have we learned and where are we going? The Andreas Grüntzig lecture ESC 2014. *Eur. Heart J.* 36 3320–3331. 10.1093/eurheartj/ehv511 26417060 PMC4677274

[B5] Colombo A. (2009). *The Role of Altered Cyclic Strain Patterns on Proliferation and Apoptosis of Vascular Smooth Muscle Cells - Implications for in-Stent Restenosis.* Ph. D. Thesis, Dublin City University, Dublin.

[B6] Dickinson R. B. Guido S. Tranquillo R. T. (1994). Biased cell migration of fibroblasts exhibiting contact guidance in oriented collagen gels. *Ann. Biomed. Eng.* 22 342–356. 10.1007/bf02368241 7998680

[B7] Dieffenbach P. B. Haeger C. M. Coronata A. M. F. Choi K. M. Varelas X. Tschumperlin D. J. (2017). Arterial stiffness induces remodeling phenotypes in pulmonary artery smooth muscle cells via YAP/TAZ-mediated repression of Cyclooxygenase-2. *Am. J. Physiol. Lung Cell. Mol. Physiol.* 313 L628–L647. 10.1152/ajplung.00173.2017 28642262 PMC5625262

[B8] Endlich N. Endlich K. Taesch N. Helwig J. J. (2000). Culture of vascular smooth muscle cells from small arteries of the rat kidney. *Kidney Int.* 57 2468–2475. 10.1046/j.1523-1755.2000.00105.x 10844615

[B9] Fomovsky G. M. Holmes J. W. (2010). Evolution of scar structure, mechanics, and ventricular function after myocardial infarction in the rat. *Am. J. Physiol. Heart Circ. Physiol.* 298 H221–H228. 10.1152/ajpheart.00495.2009 19897714 PMC2806135

[B10] Foolen J. Deshpande V. S. Kanters F. M. W. Baaijens F. P. T. (2012). The influence of matrix integrity on stress-fiber remodeling in 3D. *Biomaterials* 33 7508–7518. 10.1016/J.BIOMATERIALS.2012.06.103 22818650

[B11] Foolen J. Janssen-Van Den Broek M. W. J. T. Baaijens F. P. T. (2014). Synergy between Rho signaling and matrix density in cyclic stretch-induced stress fiber organization. *Acta Biomater.* 10 1876–1885. 10.1016/j.actbio.2013.12.001 24334146

[B12] Foolen J. Wunderli S. L. Loerakker S. Snedeker J. G. (2018). Tissue alignment enhances remodeling potential of tendon-derived cells – lessons from a novel microtissue model of tendon scarring. *Matrix Biol.* 65 14–29. 10.1016/J.MATBIO.2017.06.002 28636876

[B13] Ghazanfari S. Driessen-Mol A. Hoerstrup S. P. Baaijens F. P. T. Bouten C. V. C. (2016). Collagen matrix remodeling in stented pulmonary arteries after transapical heart valve replacement. *Cells Tissues Organs* 201 159–169. 10.1159/000442521 26989895 PMC5296926

[B14] Guido S. Tranquillo R. T. (1993). A methodology for the systematic and quantitative study of cell contact guidance in oriented collagen gels. Correlation of fibroblast orientation and gel birefringence. *J. Cell Sci.* 105(Pt 2) 317–331. 8408268 10.1242/jcs.105.2.317

[B15] Henshaw D. R. Attia E. Bhargava M. Hannafin J. A. (2006). Canine ACL fibroblast integrin expression and cell alignment in response to cyclic tensile strain in three-dimensional collagen gels. *J. Orthop. Res.* 24 481–490. 10.1002/jor.20050 16453340

[B16] Huang D. Chang T. R. Aggarwal A. Lee R. C. Ehrlich H. P. (1993). Mechanisms and dynamics of mechanical strengthening in ligament-equivalent fibroblast-populated collagen matrices. *Ann. Biomed. Eng.* 21 289–305. 10.1007/bf02368184 8328728

[B17] Jonge N. D. Kanters F. M. W. Baaijens F. P. T. Bouten C. V. C. (2013). Strain-induced collagen organization at the micro-level in fibrin-based engineered tissue constructs. *Ann. Biomed. Eng.* 41 763–774. 10.1007/s10439-012-0704-3 23184346

[B18] Lee E. J. Holmes J. W. Costa K. D. (2008). Remodeling of engineered tissue anisotropy in response to altered loading conditions. *Ann. Biomed. Eng.* 36 1322–1334. 10.1007/s10439-008-9509-9 18470621 PMC2920500

[B19] Mathieu P. S. (2020). *Multipotent Vascular Stem Cells and the Effects of Cyclic Tensile Strain, Collagen Structure and Stenting on Medial Vascular Cell Populations.* Dublin: Trinity College Dublin.

[B20] Mathieu P. S. Fitzpatrick E. Luca M. D. Cahill P. A. Lally C. (2020). Resident multipotent vascular stem cells exhibit amplitude dependent strain avoidance similar to that of vascular smooth muscle cells. *Biochem. Biophys. Res. Commun.* 521 762–768. 10.1016/j.bbrc.2019.10.185 31706573

[B21] Matsugaki A. Fujiwara N. Nakano T. (2013). Continuous cyclic stretch induces osteoblast alignment and formation of anisotropic collagen fiber matrix. *Acta Biomater.* 9 7227–7235. 10.1016/J.ACTBIO.2013.03.015 23523937

[B22] Melvin A. T. Welf E. S. Wang Y. Irvine D. J. Haugh J. M. (2011). In chemotaxing fibroblasts, both high-fidelity and weakly biased cell movements track the localization of PI3K signaling. *Biophys. J.* 100 1893–1901. 10.1016/j.bpj.2011.02.047 21504725 PMC3077704

[B23] Nolan D. R. Lally C. (2018). An investigation of damage mechanisms in mechanobiological models of in-stent restenosis. *J. Comput. Sci.* 24 132–142. 10.1016/j.jocs.2017.04.009

[B24] Peirce S. M. Van Gieson E. J. Skalak T. C. (2004). Multicellular simulation predicts microvascular patterning and in silico tissue assembly. *FASEB J.* 18 731–733. 10.1096/fj.03-0933fje 14766791

[B25] Ristori T. Notermans T. M. W. Foolen J. Kurniawan N. A. Bouten C. V. C. Baaijens F. P. T. (2018). Modelling the combined effects of collagen and cyclic strain on cellular orientation in collagenous tissues. *Sci. Rep.* 8 1–14. 10.1038/s41598-018-26989-y 29867153 PMC5986791

[B26] Rouillard A. D. Holmes J. W. (2012). Mechanical regulation of fibroblast migration and collagen remodelling in healing myocardial infarcts. *J. Physiol.* 590 4585–4602. 10.1113/jphysiol.2012.229484 22495588 PMC3477759

[B27] Sawhney R. K. Howard J. (2002). Slow local movements of collagen fibers by fibroblasts drive the rapid global self-organization of collagen gels. *J. Cell Biol.* 157 1083–1092. 10.1083/jcb.200203069 12058022 PMC2174051

[B28] Thomopoulos S. Fomovsky G. M. Holmes J. W. (2005). The development of structural and mechanical anisotropy in fibroblast populated collagen gels. *J. Biomech. Eng.* 127:742. 10.1115/1.1992525 16248303

[B29] Wang J. H. C. Jia F. Gilbert T. W. Woo S. L. Y. (2003). Cell orientation determines the alignment of cell-produced collagenous matrix. *J. Biomech.* 36 97–102. 10.1016/S0021-9290(02)00233-6 12485643

[B30] Yi B. Shen Y. Tang H. Wang X. Li B. Zhang Y. (2019). Stiffness of aligned fibers regulates the phenotypic expression of vascular smooth muscle cells. *ACS Appl. Mater. Interfaces* 11 6867–6880. 10.1021/acsami.9b00293 30676736

[B31] Zahedmanesh H. Lally C. (2012). A multiscale mechanobiological modelling framework using agent-based models and finite element analysis: application to vascular tissue engineering. *Biomech. Model. Mechanobiol.* 11 363–377. 10.1007/s10237-011-0316-0 21626394

[B32] Zahedmanesh H. Van Oosterwyck H. Lally C. (2014). A multi-scale mechanobiological model of in-stent restenosis: deciphering the role of matrix metalloproteinase and extracellular matrix changes. *Comput. Methods Biomech. Biomed. Eng.* 17 813–828. 10.1080/10255842.2012.716830 22967148

[B33] Zhou N. Stoll S. Leimena C. Qiu H. (2018). “Vascular smooth muscle cell,” in *Muscle Cell and Tissue - Current Status of Research Field*, ed. Sakuma K. (London: InTech), 10.5772/intechopen.77249

[B34] Zhu J. H. Chen C. L. Flavahan S. Harr J. Su B. Flavahan N. A. (2011). Cyclic stretch stimulates vascular smooth muscle cell alignment by redoxdependent activation of notch3. *Am. J. Physiol. Heart Circ. Physiol.* 300 H1770–H1780. 10.1152/ajpheart.00535.2010 21169401 PMC3094076

