# Supplementary material for: An in-silico Investigation Into the Role of Strain and Structure on Vascular Smooth Muscle Cell Growth
Source: Front Bioeng Biotechnol. 2021 Apr 20;9:641794. doi: 10.3389/fbioe.2021.641794 (PMC8093633; doi:10.3389/fbioe.2021.641794)
Supplement: Supplementary file 1 [file Data_Sheet_1.docx]

Supplementary Material

# Experimental Cell Reorientation Data
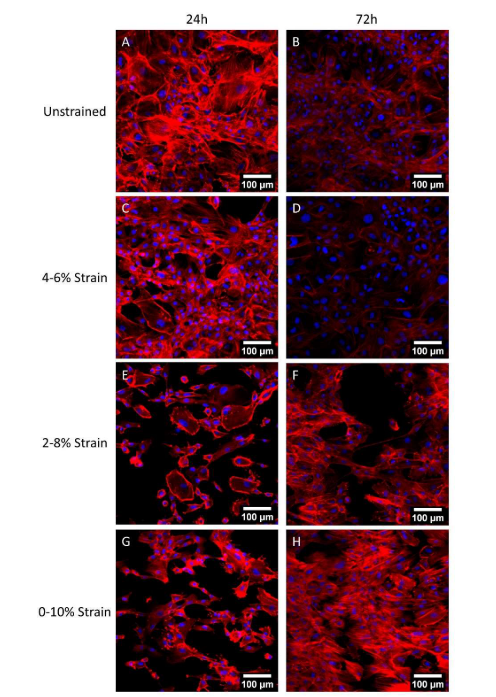


# Figure 1. Representative images of RASMC after 24 (A, C, E, G) or 72 hours (B, D, F, H) of no strain (A, B), or 4-6% (C, D), 2-8% (E, F), or 0-10% (G, H) 1 Hz cyclic tensile strain. Blue – DAPI nuclei, Red – Phalloidin f-actin (Mathieu 2020).


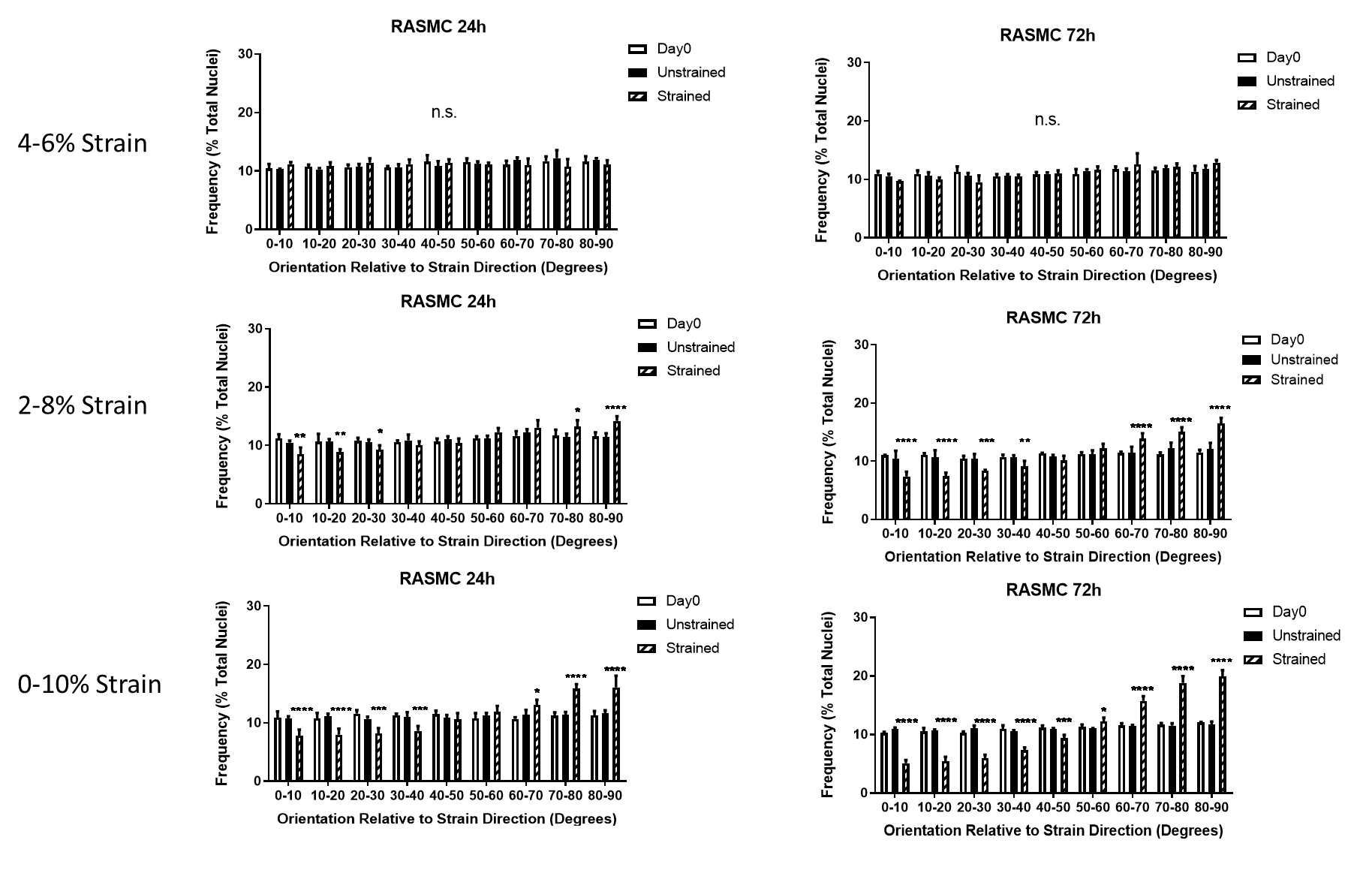


**Figure 2.** Experimental cell reorientation data displaying the percentage of nuclei and their orientation in relation to strain at 24 and 72hr when the cells are subject to 4-6%, 2-8% and 0-10% cyclic strain (Mathieu 2020).

# Cell Radius ($\boldsymbol{R}_{\boldsymbol{c}}$)

As confluence was not seen in the experimental data the following sensitivity analysis was performed on the cell radius to ensure that it was not the influencing parameter in predicting the final cell density due to an artificial over confluence. The example of an initial cell density of 305 cell/mm^2^ was used. Firstly, the model was run for different iterations using the same parameters ($R_{c}$ of 12.96 μm (Zahedmanesh and Lally 2012; Zahedmanesh, Van Oosterwyck, and Lally 2014)) to investigate the influence of the inherent randomness in the model. A change in the cell density of 1-2% was deemed acceptable. Different magnitudes of $R_{c}$ were then examined and it was found that a value of $R_{c}$ of 0.3888 μm ($R_{c}x0.03$ ) was appropriate in predicting cell density (Table 1).

**Table 1.** Sensitivity of Cell Density Prediction to $R_{c}$

| Magnitude of $\boldsymbol{R}_{\boldsymbol{c}}$ | Predicted Day 3 Cell Density | Difference for Previous $\boldsymbol{R}_{\boldsymbol{c}}$Value (%) |
| --- | --- | --- |
| $R_{c}=12.96$ (iteration 1) | 608 |  |
| $R_{c}=12.96$ (iteration 2) | 613 | 0.82 |
| $R_{c}x10$ | 171 | -72.10 |
| $R_{c}x2$ | 228 | 33.33 |
| $R_{c}x1.5$ | 341 | 49.56 |
| $R_{c}x1.2$ | 478 | 40.18 |
| $R_{c}x0.5$ | 947 | 98.12 |
| $R_{c}x0.1$ | 972 | 2.64 |
| $R_{c}x0.05$ | 966 | -0.62 |
| $R_{c}x0.03$ | 1014 | 4.97 |
| $R_{c}x0.02$ | 1006 | -0.79 |
| $R_{c}x0.01$ | 1002 | -0.40 |
| $R_{c}x0.001$ | 1016 | 1.40 |

# Influence of Dispersion, κ, on Generated Fibre Distribution


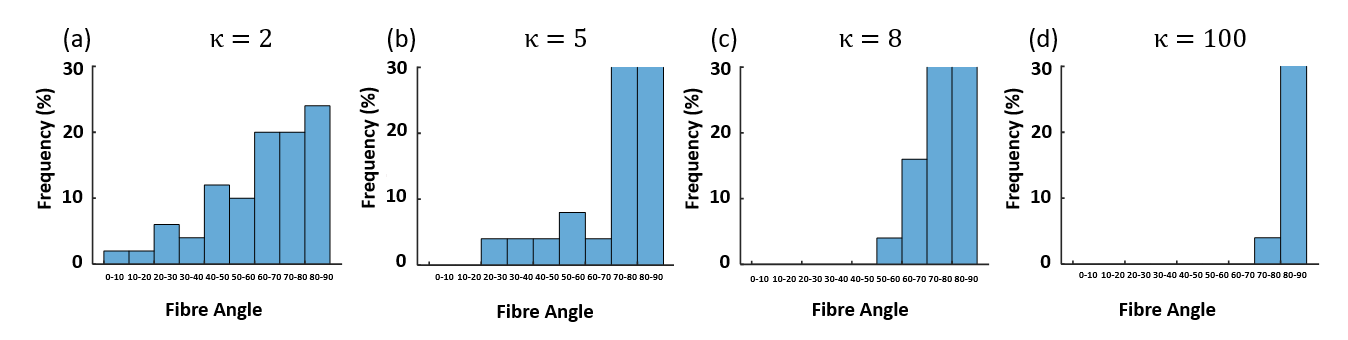


**Figure 4.** Fibre distributions demonstrating influence of dispersion with (a) κ=2 (b) κ=5 (c) κ=8 (d) κ=100.

# Sensitivity Analysis

The sensitivity of the predicted results to each of the parameters was investigated. The parameters used to predict change in cell density in the absence of external stimulus were examined first. This was done by varying each parameter individually by ± 10% and keeping the other parameters constant ($C_{prolif}=71$, $\sigma_{prolif}=9$, $B_{apop}=1.64$) to examine how cell behaviour changed.

**Table 2.** Influence of $C_{prolif}$, $\sigma_{prolif}$, and $B_{apop}$

| Parameter | x0.9 | x 1 | x1.1 |
| --- | --- | --- | --- |
| $C_{prolif}$ | 923.90 | 763.38 | 644.36 |
| $\sigma_{prolif}$ | 731.68 | 763.38 | 804.96 |
| $B_{apop}$ | 842.48 | 763.38 | 695.48 |

The sensitivity of the model to these parameters can be seen in Table 2. Increasing $C_{prolif}$ by 10% led to a 16% decrease in cell density whereas a 10% decrease led to a 21% increase in cell density after 3 days. When $B_{apop}$ was increased by 10% the model predicted a 10% increase in cell density and decreased 9% with a 10% decrease. Conversely, a 10% increase and decrease in $\sigma_{prolif}$ led to a 5% increase and a 4% decrease in cell density, respectively.

A sensitivity analysis was also performed on the parameters relating to the influence of cyclic strain. The sensitivity of each of the three parameters was investigated by increasing and decreasing the value by an order of magnitude and investigating how the fold change in cell number was influenced, see Table 3.

**Table 3.** Influence of $A_{prolif,} A_{apop}$ and $B_{prolif}$ on fold change in cell number

| 0-10% | 24 Hr | 72 Hr |
| --- | --- | --- |
| Experimental | 0.80 ± 0.14 | 0.939 ± 0.12 |
| Original Parameters | 0.87 | 0.83 |
| Aprolif x10 | 0.77 | 0.54 |
| Aprolif x0.1 | 0.94 | 0.99 |
| Bprolif x10 | 0.69 | 0.38 |
| Bprolif x0.1 | 1.12 | 1.14 |
| Aapop x10 | 0.79 | 0.62 |
| Aapop x0.1 | 0.89 | 0.85 |

The influence of $\varepsilon_{Max}$ and $\varepsilon_{Thres}$on fold change were investigated and can be seen in Table

**Table 4.** Influence of $\varepsilon_{Max}$ and $\varepsilon_{Thres}$ on Fold Change in Cell Number

| 0-10% | 24 Hr | 72 Hr |
| --- | --- | --- |
| Experimental | 0.80 ± 0.14 | 0.939 ± 0.12 |
| Original Parameters | 0.87 | 0.83 |
| $\varepsilon_{Max}$ x10 | 0.88 | 0.81 |
| $\varepsilon_{Max}$ x0.1 | 0.89 | 0.86 |
| $\varepsilon_{Thres}$ (+0.05) | 0.87 | 0.82 |
| $\varepsilon_{Thres}$ (+0.5) | 0.93 | 0.94 |
